# Patterns of Metastatic Recurrence of Genetically Confirmed Myxoid Liposarcoma

**DOI:** 10.1245/s10434-023-13312-x

**Published:** 2023-03-12

**Authors:** Pauliina Homsy, Tom Böhling, Anne Seitsonen, Mika Sampo, Erkki Tukiainen, Carl Blomqvist

**Affiliations:** 1 Department of Plastic Surgery, University of Helsinki and Helsinki University Hospital, Helsinki, Finland; 2 Department of Pathology, University of Helsinki and Helsinki University Hospital, Helsinki, Finland; 3 HUS Diagnostic Center, Helsinki University Hospital, Helsinki, Finland; 4 Department of Oncology, University of Helsinki and Helsinki University Hospital, Helsinki, Finland

## Abstract

**Background:**

Most sarcomas metastasize predominantly to the lungs, and chest x-ray, or computed tomography, is the most commonly used staging investigation. Myxoid liposarcomas (MLSs) are rare tumors with a tendency to metastasize to extrapulmonary loci. The aim of this study was to assess the locations of the first metastases in MLS patients, to guide the design of effective staging and follow-up imaging protocols.

**Methods:**

Patients treated for MLS between 1987 and 2017 were identified in a prospectively maintained register. Histology of the tumors was reassessed. In addition, the presence of one of the pathognomonic gene translocations was confirmed, uniquely for a retrospective series. The surgical and oncological outcomes were reviewed. A comprehensive review of the literature was performed on the metastatic pattern of MLS, including series with 10 or more MLS patients with metastatic disease.

**Results:**

A total of 32 patients with genetically confirmed MLS were identified, with a median follow-up of 7.6 years. Seven patients (22%) developed metastatic disease, five initially intra-abdominally and only one to the lungs. The comprehensive review included 14 series with 1853 patients, 348 (19%) of whom had metastases. The location of the first metastases was soft tissues in 32% of patients, intra-abdominal in 26%, pulmonary in 24%, and bone in 17%.

**Conclusions:**

MLSs metastasize often intra-abdominally and to extra-abdominal soft tissues. Thus, whole-body imaging may be indicated during the initial assessment and follow-up of these patients.

**Supplementary Information:**

The online version contains supplementary material available at 10.1245/s10434-023-13312-x.

Myxoid liposarcomas (MLSs) are rare tumors, accounting for around one-third of liposarcomas and 10% of soft tissue sarcomas.^1^ Four distinct histological subtypes of liposarcoma have been identified: well-differentiated, dedifferentiated, myxoid, and pleomorphic.^1^ The myxoid variety has a peak presentation 10 years earlier than the other subtypes, early in the fifth decade.^2^ In addition, while being highly radiosensitive and rarely recurring locally, MLSs have a strong tendency to metastasize.^2–5^ Thus, tailoring the oncological follow-up of these patients in a way that reflects the likely pattern of disease recurrence is indicated.

Identification of the MLSs has historically relied on their histological appearance with the myxoid stroma and an arborating capillary network. Two pathognomonic gene translocations have been identified, with the more common t(12;16)(q13;p11) resulting in fusion of FUS and DDIT3, previously known as TLS and CHOP, respectively, and the t(12;22)(q13;q12) translocation resulting in *EWSR1:DDIT3* fusion.^6–12^ The presence of the fusion protein has been suggested to inhibit adipocyte differentiation.^13^ The specific type of the translocation has not been shown to predict the course of the disease.^14^ While many centers now routinely use gene panel analysis for the diagnosis of soft tissue tumors, the requirement for a genetic analysis is not yet included in most treatment guidelines.

The tendency of MLSs to develop extrapulmonary metastases is well-documented.^15–18^ However, previous series contain patients who do not have the genetic diagnosis confirmed or reported. In addition, the detailed pattern of extrapulmonary metastasis is less established. It is questionable, whether the practice of imaging only the primary tumor location and chest, as done with other soft tissue sarcomas during staging and follow-up, is sufficient in MLS patients.

The aim of this study was to assess the locations of MLS metastases in our series of genetically confirmed MLS patients, and in the existing MLS literature, to enable evaluation of whether whole-body imaging at primary staging and during follow-up is likely to improve the detection of metastases compared with chest imaging alone.

## Methods

Patients treated for MLS by the multidisciplinary sarcoma team of Helsinki University Hospital between 1987 and 2017 were identified using a prospectively maintained sarcoma database and a pathology database. Patients for whom patient records and tissue samples were available were included for initial review. The tumor histology was reassessed. Those filling the histological criteria for MLS were subjected to genetic analysis.

The presence of the most common gene fusion pathognomonic of myoxid liposarcoma, *FUS::DDIT3* was evaluated from paraffin-embedded tissue samples with fluorescent in situ hybridization (FISH) using an FUS (16p11) dual-color break-apart probe according to the manufacturer’s (Vysis, Abbott) protocol.^19^ Tumors that did not have the index translocation detectable on FISH were further analyzed with the Archer FusionPlex Sarcoma panel that assesses 148 locations from RNA using next-generation sequencing (NGS).^20^ Only tumors that displayed the *FUS::DDIT3* translocation or the *ESWRT1::DDIT3* fusion were included in the final study population.

Of the 46 patients identified in the databases and classified as MLSs in the histology review, FISH analysis showed an *FUS* translocation, suggesting *FUS::DDIT3* fusion, in 26 tumors. No translocation was identified in 11 samples and 9 of the FISH analyses produced no result. The NGS sarcoma panel was run on these 20 samples, revealing *FUS::DDIT3* fusion in four samples and *ESWR1::DDIT3* fusion in two samples. No result was obtained in six of the samples and eight of the samples did not reveal either one of the pathognomonic translocations. Two of these patients had other translocations: *NAB2::STAT6* fusion and *COL1A1::PDGFB* fusion, suggestive of solitary fibrous tumor and dermatofibrosarcoma protuberans, respectively.^21,22^ Thus, the final study population was 32 patients who filled the criteria of having genetically confirmed MLS.

The histology was reviewed for tumor grading according to the French Federation Nationale des Centers de Lutte Contre le Cancer.^23^ The round cell variant MLS tumors were classified as grade 3. The original pathology reports were used for the tumor size and resection margins, defined as R0 = microscopically negative margins, R1 = microscopically positive margins, and R2 = macroscopically positive margins. The patient records were reviewed for the demographic and follow-up details. All primary MLS tumors treated with curative intent are surgically removed. In our institute, radiotherapy is offered preoperatively for tumors for which the resection is predicted to result in macroscopically or microscopically positive margins, while postoperative radiotherapy is recommended after marginal or intralesional surgery. Adjuvant chemotherapy with six courses of doxorubicin ifosfamide is recommended for patients with a good performance status and a high-grade tumor fulfilling at least two of the following criteria: size larger than 8 cm, necrosis or vascular invasion.^24^ Recurrence-free patients are followed-up for 5 (high-grade) or 10 years (low-grade) postoperatively. Regular imaging of the primary site and lungs are performed during follow-up. No whole-body imaging is performed. The first and subsequent locations of the metastases were recorded.

Kaplan–Meier analysis was used for the calculation of metastases-free survival, local recurrence-free survival, and disease-specific survival. IBM SPSS Statistics 27 software (IBM Corporation, Armonk, NY, USA) was used for all the analyses.^25^

The study was approved by the Helsinki University research Ethics Committee, license number 270/12/03/2011, and the Finnish Institute for Health and Welfare, license number THL/1303/5.05.00/2011.

### Literature Review

The pattern of MLS metastases locations was assessed through a comprehensive review of literature following the Preferred Reporting Items for Systematic Reviews and Meta-Analyses (PRISMA) guidelines.^26^ A systematic literature search of the MEDLINE, Web of Science and Scopus databases was performed on 22 June 2022 using the search terms shown in Table 1. Articles including at least 10 patients with MLS metastases were assessed for eligibility and those reporting the locations and sequence of metastases in the patients were included (electronic supplementary material [ESM] 1)

**Table 1:** Search terms for the literature review run on 24 June 2022

Database	Search terms
MEDLINE	(liposarcoma myxoid) AND ((neoplasm metastasis) OR metastasis)
Web of Science Core Collection	**TOPIC:** (myxoid* near/1 liposarcoma*) *AND* **TOPIC:** (metasta*)
Scopus	TITLE-ABS-KEY (liposarcoma myxoid) AND ((neoplasm metastasis) OR metastasis) AND NOT INDEX (MEDLINE)

Location of the first metastasis was recorded for each of the patients in the included series. The locations were classified as pulmonary, intra-abdominal, extra-abdominal soft tissue, bone, or multiple. Abdominal solid organ metastases and retroperitoneal metastases were included in the intra-abdominal group, and lymph node metastases were included in the soft tissue group. Pulmonary and pleural metastases were reported together. The proportion of the metastases in each location among metastatic cases in each series was calculated and summarized for the whole review population. The Clopper–Pearson 95% confidence intervals (CIs) were calculated using Epitools.^27^

## Results

The final study group included 32 patients with MLS—21 men and 11 women. The median age at diagnosis was 47.7 years (range 22.7–82.7 years). Twenty-one of the tumors were grade 2 and 11 were grade 3. The location of the primary tumor was the lower limb in 29 patients, and in the upper limb, the head and the trunk wall for one patient each. The maximum diameter of the tumor was under 10 cm in 14 (44%) patients and 10 cm or over in 17 (53%) patients. One of the primary surgeries had been performed elsewhere in 1987, and the information of the size of the primary tumor was unavailable. The resection margins were R0 in 8 (25%) patients, R1 in 19 (59%) patients, and R2 in 3 (9%) patients. Data on resection margins were not available for two of the patients. Twenty patients (63%) received radiotherapy during the primary presentation, including all patients with R2 resection, no patients with R0 resection, and four (13%) patients with chemotherapy. The median follow-up time was 7.6 years (range 2.1–16.7).

During the follow-up, six (19%) patients developed local disease progression, including one of the patients with R2 resection and radiotherapy and both patients with currently unknown resection margins. Three of the patients with a local recurrence had an R1 resection margin, two with adjuvant radiotherapy. No tumor with R0 margin recurred locally. Four of the local recurrences were in the lower limb, one in the upper limb, and one in the trunk wall. The 5-year local disease progression-free survival was 84 ± 7% (ESM 2).

**Fig. 1:**
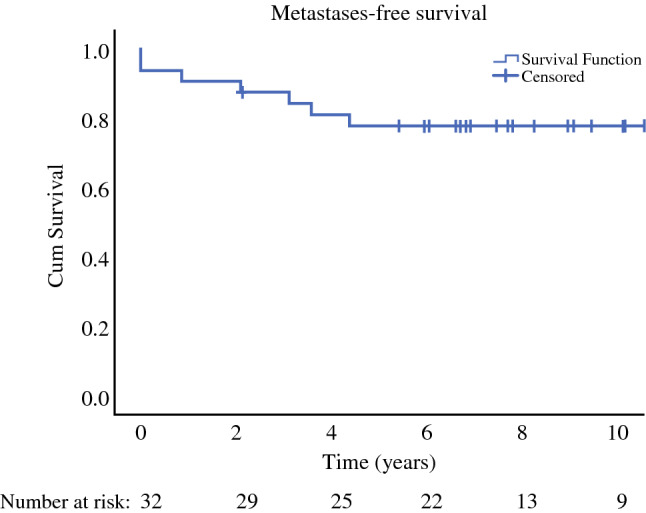
Metastases-free survival of patients with myxoid liposarcoma in the current series. *Cum* cumulative

Metastases were detected in seven patients (22%), including two of the patients (6%) presenting primarily with metastatic disease (Table 2). The 5-year metastases-free survival was 78 ± 7% (Fig. 1). Each of the patients initially had a single metastatic location. Locations of the metastases were as follows: Patient 1: lungs and pleura, operated and recurring later in the lungs; Patient 2: first in the abdomen and in a supraclavicular lymph node, later in the subcutaneous tissue, again in the abdomen, lungs and spine; Patient 3: in the abdomen; Patient 4: first in the abdomen, later again in the abdomen as well as subcutaneous tissues around the scar; Patient 5: the brain; Patient 6: in the abdomen and contralateral thigh soft tissue; and Patient 7: first in the abdomen, later in the bone. Four of these patients also developed disease recurrence in the primary tumor location.

**Table 2:** Myxoid liposarcoma patients with metastatic disease

Patient	Site of primary tumor	Maximum diameter of primary tumor (cm)	Primary resection margins^a^	Radiotherapyfor the primary tumor?	Local recurrence?	Location of first metastases	First disease recurrence (months)
1	Lower limb	20	R2	Yes	Yes	Lungs and pleura	0^b^
2	Lower limb	7	NA	No	Yes	Abdominal cavity, supraclavicular lymph node	0^c^
3	Lower limb	4	R0	No	No	Abdominal cavity	37
4	Lower limb	30	R1	Yes	Yes	Abdominal cavity	10^d^
5	Lower limb	28	R1	Yes	No	Brain	52
6	Lower limb	23	R1	Yes	No	Abdominal cavity and contralateral thigh soft tissue	25
7	Axilla	7	NA	Yes	Yes	Abdominal cavity	35^e^

*NA* not available

^a^R0 = microscopically negative margins, R1 = microscopically positive margins, R2 = macroscopically positive margins

^b^Metastases at presentation, resected and recurred at 12 months

^c^Metastases at presentation, not all resected

^d^Both local recurrence and metastases detected at 10 months

^e^Local recurrence at 35 months, metastases at 43 months

Overall, nine patients had a local recurrence or metastases during follow-up, with a 5-year disease-free survival of 75 ± 8%. Six patients died of MLS, with a 5-year disease-specific survival of 87 ± 6%.

### Literature Review

Seventeen articles describing unique patient series with at least 10 patients with metastatic MLS were identified;^2,4,5,15,17,18,28–37^ however, two of the articles classified metastasis locations as only ‘pulmonary’ or ‘extrapulmonary’ and were thus excluded from the analysis.^15,37^ In addition, one of the articles did not enable complete separation between the first and subsequent metastases and was excluded.^36^ The series included a total of 1853 patients with MLS, 348 (19%) of whom had metastatic disease (Table 3). The gene translocation status was not reported for any of the review patients.

**Table 3:** Series of patients with myxoid liposarcoma metastases included in the meta-analysis

Author	Publication year	City	Time period	Number of patients with myxoid liposarcoma	Number of patients with metastases	Follow-up time (months, median)
Smith et al.^28^	1996	Cleveland, USA	1961–1992	29	10	72
Zagars et al.^2^	1996	Houston, USA	1964–1992	71	13	109^a^
Spillane et al.^29^	1999	London, UK	1988–1998	50	12	43
ten Heuvel et al.^30^	2007	Groningen, Holland	1977–2004	49	13	101
Sheah et al.^31^	2008	Massachusetts, USA	1999–2007	112	12	Not reported
Guadagnolo et al.^5^	2008	Houston, USA	1960–2003	127	27	109
Chung et al.^4^	2009	Toronto, Canada	1986–2004	88	12	86
Haniball et al.^18^	2011	Birmingham, USA	1987–2005	160	52	55
Moreau et al.^17^	2012	Montreal, Canada	1977–2008	418	83	62
Hoffman et al.^32^	2012	Houston, USA	1990–2010	207	26	68
Baxter et al.^33^	2015	Atlanta, USA	2000–2010	75	10	60
Muratori et al.^16^	2018	Firenze, Italy	1994–2015	148	20	63^b^
Gouin et al.^34^	2019	Lyon, France	2006–2011	45	10	43
Shinoda et al.^35^	2020	Tokyo, Japan	2001–2015	274	48	47

^a^Time for all types of liposarcoma included in the article

^b^Average follow-up time

**Fig. 2:**
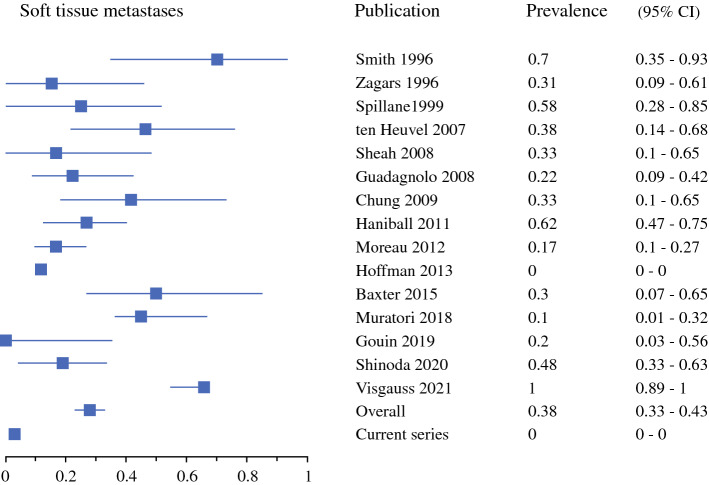
First metastatic recurrence in soft tissues in myxoid liposarcoma patients. *CI* confidence interval

**Fig. 3:**
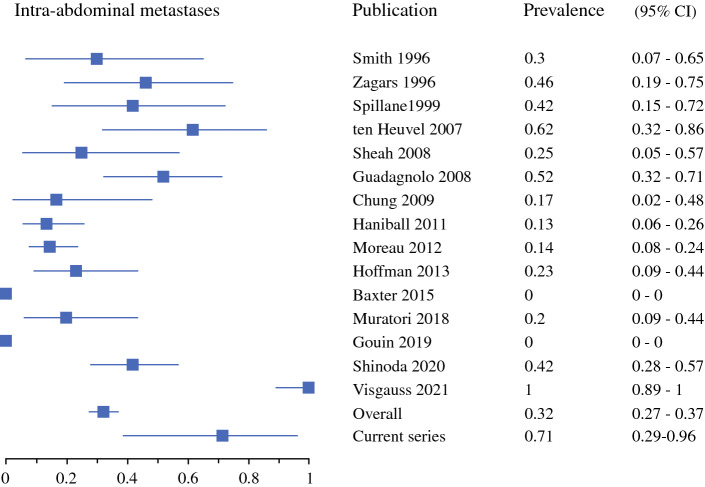
First metastatic recurrence intra-abdominally in myxoid liposarcoma patients. *CI* confidence interval

**Fig. 4:**
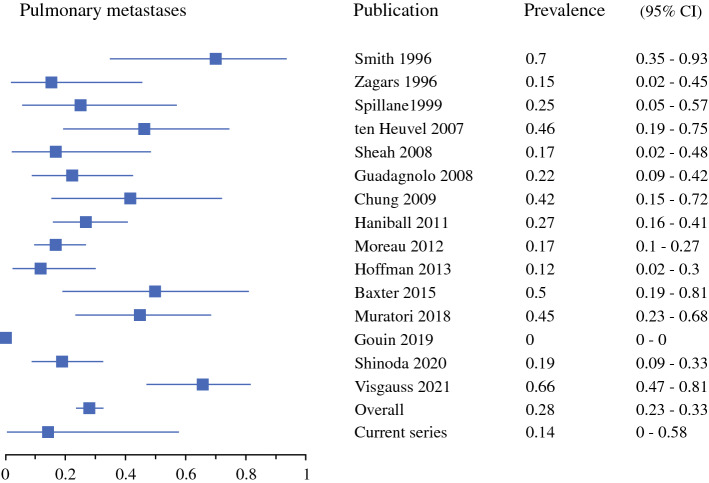
First metastatic recurrence in the lungs in myxoid liposarcoma patients. *CI* confidence interval

**Fig. 5:**
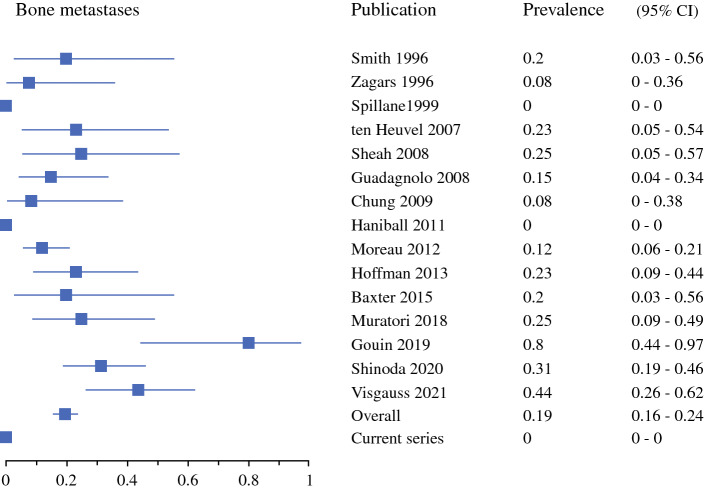
First metastatic recurrence in bone in myxoid liposarcoma patients. *CI* confidence interval

Soft tissue metastases were most common, with a 32% prevalence (95% CI 28–38%) [Fig. 2], followed by intra-abdominal at 26% (95% CI 21–31%) [Fig. 3], pulmonary at 24% (95% CI 19–29%) [Fig. 4], and bone metastases at 17% (95% CI 13–22%) [Fig. 5]. Of note, for 12 of the series, pulmonary and pleural metastases were reported together, or the potential inclusion of pleural metastases in the lung metastases counts was not disclosed.^4,5,16–18,30,32–36,38^ Primary metastases were detected at multiple locations in 26% (95% CI 22–31%).

## Discussion

MLS often has a fairly good prognosis, with a 10-year disease-specific survival of 56–87% reported in different series.^2,5,16,18,30,32^ A population-based Dutch study of 901 cases of MLS reported 5- and 10-year overall survival rates of 78 and 66%, respectively.^39^ High tumor grade with large round cell percentage has been associated with reduced disease-specific survival.^14,18,40–42^ The development of metastases bears a worse prognosis and the frequency of metastatic disease both at presentation and during follow-up is higher for myxoid than for other histological subtypes of liposarcoma.^2,29,43^ By 5 years from diagnosis, metastatic disease has been detected in 15–44% of patients.^5,16,18,34^ Understanding the pattern of metastases of MLS is important in guiding follow-up imaging with the aim of detecting symptomless metastases.

To our knowledge, our patient population is the first series to include exclusively MLS patients with a confirmed pathognomonic gene translocation. The 5-year disease-free survival was 75% and the disease-specific survival was 82%. Overall, the disease course observed for these patients is similar to that reported by other centers with series containing patients for whom the diagnosis of MLS was based on histology alone.

The predominance of lower limb primary tumors (91% in our series) is in line with the figures reported elsewhere (78–96%).^2,5,16–18,29,30,33,34^ This is notably more than for other types of soft tissue sarcomas as fewer than one-third of all primary sarcomas occur in the lower extremity.^44^ The proportion of first metastases in the abdominal cavity was higher in our series than in those included in the literature review. However, further studies are needed to assess whether genetically confirmed MLSs truly have stronger tendency for abdominal metastases than previously reported.

The stringent inclusion criteria applied to our series may have resulted in an exclusion of some of the patients with degraded tumor samples or with gene fusion variants not detected by the sequencing probes. However, our retrospective series reflect the current diagnostic criteria for MLS. An interesting observation was that two of the tumors had gene translocations suggestive of another tumor diagnosis, normally encountered in solitary fibrous tumor and dermatofibrosarcoma protuberans. Thus, it is possible that similar outliers are included in earlier published series.

A 5-year metastases-free survival of 78% was observed in our patient series. Of the seven patients with metastases, five had abdomen as the first location of metastases and only one patient had lung metastases combined with pleural disease as the first location of metastases. None of the lung metastases were resectable. Extrapulmonary metastases have previously been recognized to occur more commonly in MLS than other liposarcoma types.^3–5,15^ Of the 1853 MLS patients included in the present review, only 24% had first metastases confined to the lungs or pleura. Isolated lung recurrence is probably even less frequent since most of the studies reported lung and pleural metastases together. The metastases were first detected in the soft tissues in 32%, intra-abdominally or retroperitoneally in 26%, and in bone in 17%. Several metastases were discovered simultaneously in 26%. In our series, none of the seven metastatic cases had a first relapse exclusively to the lungs. Thus, this review reaffirms the tendency of MLS to develop extrapulmonary metastases. No association was observed between the location of the primary tumor and the first metastases in our patients or the review series, pointing to likely hematologic spread, independent of vascular anatomy.^28–31^

The high prevalence of intra-abdominal, including retroperitoneal, metastases is interesting as primary retroperitoneal MLSs are rare.^45,46^ Suspected primary retroperitoneal tumors have been suggested to be either metastases from an undetected primary or well-/dedifferentiated liposarcomas with prominent myxoid degeneration.^46,47^ Two of the series in the review included patients with the primary tumor located in the retroperitoneum;^5,30^ however, both of the series relied on the histological diagnosis of the tumors without genetic confirmation, and neither reported on whether imaging was performed for metachronous tumors elsewhere in the body.

Contemporary guidelines (European Society for Medical Oncology [ESMO] 2018 ja 2021) recommend imaging of the primary tumor site and the lungs for the majority of soft-tissue sarcomas, since the lungs are usually the first and most common site of metastases. For some histological subgroups, including MLS, abdominal or whole-body imaging is also proposed. For follow-up, regular imaging of the primary site and the chest is recommended in order to diagnose relapses, which are potentially curable with surgery as soon as possible (ESMO 2018). Resection of pulmonary metastases with tumor-free margins is potentially curative in patients with soft tissue sarcoma.^48^ The surgery is an option for patients with sufficient performance status and no active disease at the primary tumor site. In addition, the metastatic nodules need to be confined to the lung parenchyma and be either singular or distributed in a manner that enables complete surgical resection. The presence of a single metastatic lesion is associated with improved survival.^49,50^ Incomplete surgical removal of pulmonary metastases does not convey better disease-free survival than chemotherapy.^48,50^ While these studies contain patients with a range of soft tissue sarcomas, the resection of lung metastases should be considered an option for patients with MLS, supporting the use of pulmonary imaging in the follow-up of these patients.

With pulmonary metastases being the first presentation of metastatic disease in less than one-third of patients with MLS, clinicians are faced with the challenge of determining the most appropriate imaging for these patients for primary staging and follow-up. Inclusion of abdominal CT scans in the staging and follow-up imaging of patients with MLS has been proposed.^4,15^ In light of the propensity of MLS to develop soft tissue metastases, whole-body imaging may also reveal that a tumor, initially regarded as primary, is in fact a likely metastases from a previously undetected primary. Conventional abdominal CT scans involve significant radiation exposure, and, focusing on the abdomen, the scans fail to capture soft tissue metastases in the limbs, chest and head and neck regions. The ability of a CT scan, a positron emission tomography (PET)/CT scan, or a bone scan to detect MLS bone metastases has also been questioned.^36,51^ In particular, the negative predictive power of a bone scan and a PET scan in detecting spinal metastases has been suggested to be only 88% and 85%, respectively.^51^ Furthermore, the proportion of MLS bone metastases that are sclerotic, and thus easily detectable by CT, could be as low as <10%.^36^

Whole-body magnetic resonance imaging (MRI) scans have been proposed as a suitable imaging modality in the follow-up of MLS patients.^52^ MRI scans have also been proposed to have higher sensitivity for bone metastases of MLS than CT or bone scans.^36,51^ However, whole-body MRI scans are resource-intensive and not part of the standard follow-up regimen for liposarcomas in many centers, including ours.^2,4,5,16–18,25,28–33,35,36^ While only two of the centers behind the series included in the present review use whole-body MRI in the follow-up of MLS patients,^34,36^ 4 of the 15 authors recommend its introduction into the follow-up protocol.^16,30,34,36^ Two additional authors recognize its value for patients with already metastatic disease or localized disease with high round cell percentage, and therefore a presumed increased risk of developing metastases.^17,18^ However, no studies have yet demonstrated an improved disease-specific survival from the earlier detection of the metastases that whole-body MRI might bring. In our institute, the use of whole-body MRI is reserved for the surveillance of patients with a genetic mutation known to increase the risk of a variety of cancers, such as those with Li-Fraumeni syndrome.

Our study is limited by the retrospective nature of our series and those included in the review. In addition, many studies were small, and only 14 series with 10 or more MLS patients with metastatic disease and sufficient data regarding the location of the metastases were identified. However, the total number of MLS patients included in the review, i.e. 1853 overall and 348 with metastases, is reasonable. Unfortunately, most of the studies in the review did not allow the estimation of the proportion of patients with lung metastases suitable for surgical resection.

A distinct feature of our series is the examination of the tumor samples, not only for histological diagnosis but also for the presence of the pathognomonic gene translocation *FUS::DDIT3* or *EWSR1::DDIT3*. Of the 46 MLS tumors identified based on the histology, 14 were excluded from the final series based on the gene analysis. Six of the samples were too degraded to produce a result on either FISH analysis or NGS, and eight of the tumors did not have either of the gene fusions. The presence of the pathognomonic fusion gene was not an inclusion criterium for any of the retrospective series and was not consistently reported.^2,4,5,16–18,28–36^

## Conclusion

Data from our hospital and other patient series suggest that metastatic disease in MLS is common. The most common site of the metastases is soft tissues, followed by the abdomen and lungs. Thus, imaging of only the primary tumor site and lungs during follow-up is insufficient at detecting the majority of the metastases, and is thus inadequate for primary staging. However, more studies are needed to assess whether the introduction of whole-body imaging to the follow-up regimen of MLS patients could improve their prognosis.

## Supplementary Information

Below is the link to the electronic supplementary material.

Supplementary file1 (DOCX 60 kb)

Supplementary file2 (JPG 24 kb)

Supplementary file3 (JPG 22 kb)

Supplementary file4 (JPG 23 kb)

Supplementary file5 (DOCX 11 kb)
