# Supplementary material for: Patterns of Metastatic Recurrence of Genetically Confirmed Myxoid Liposarcoma
Source: Ann Surg Oncol. 2023 Mar 12;30(7):4489–97. doi: 10.1245/s10434-023-13312-x (PMC10250512; doi:10.1245/s10434-023-13312-x)
Supplement: Supplementary file 1 — Supplementary file1 (DOCX 60 kb) [file 10434_2023_13312_MOESM1_ESM.docx]

**Identification of studies via databases and registers**

**Identification of studies via other methods**

Records removed *before screening*:

Duplicate records removed (n = 189 )

Records identified from:

Citation searching (n = 8)

Records identified from Databases:

PubMed (n = 258)

Web of Science (n =336)

Scopus (n = 95)

**Identification**

Records excluded for not including at least ten patients metastatic myxoid liposarcoma (n = 463)

Records screened

(n = 500)

Reports not retrieved

(n = 0)

Reports not retrieved

(n = 0)

Reports sought for retrieval

(n = 8)

Reports sought for retrieval

(n = 37)

**Screening**

Reports excluded:

Lists metastases only as “pulmonary” or “extrapulmonary” (n = 2)

Primary and subsequent metastases not distinguishable (n = 1)

Focuses only on extrapulmonary metastases (n = 2)

Study population includes only advances disease (n = 1)

Metastases sites not listed (n = 20)

Reports assessed for eligibility

(n = 8)

Reports excluded:

Does not meet the inclusion criteria (n = 3)

Data included also in another report (n = 2)

Reports assessed for eligibility

(n = 37)

Studies included in the review

(n = 14)

**Included**
