# Supplementary figures and images for: Patterns of Metastatic Recurrence of Genetically Confirmed Myxoid Liposarcoma
Source: Ann Surg Oncol. 2023 Mar 12;30(7):4489–97. doi: 10.1245/s10434-023-13312-x (PMC10250512; doi:10.1245/s10434-023-13312-x)

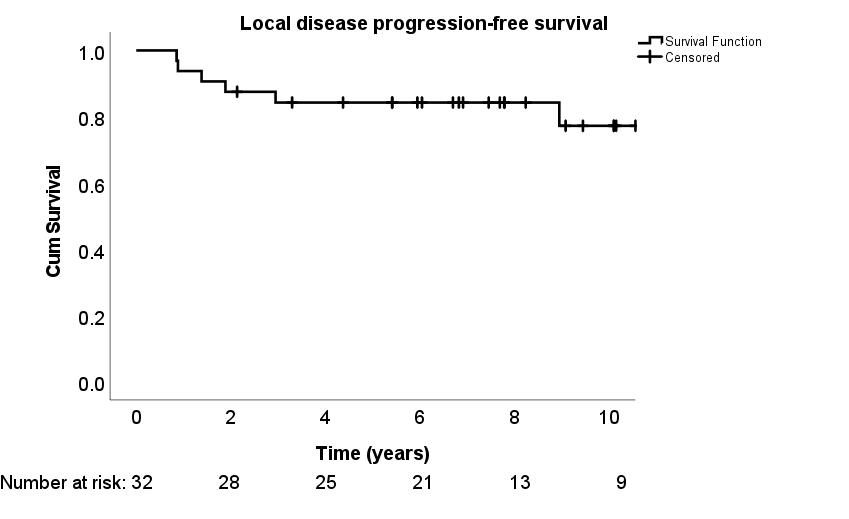

Supplement: Supplementary file 2 — Supplementary file2 (JPG 24 kb) [file 10434_2023_13312_MOESM2_ESM.jpg]

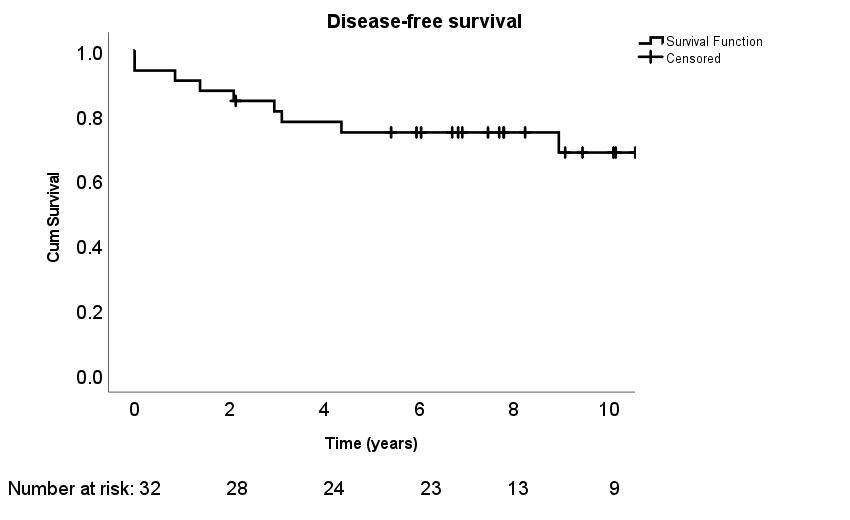

Supplement: Supplementary file 3 — Supplementary file3 (JPG 22 kb) [file 10434_2023_13312_MOESM3_ESM.jpg]

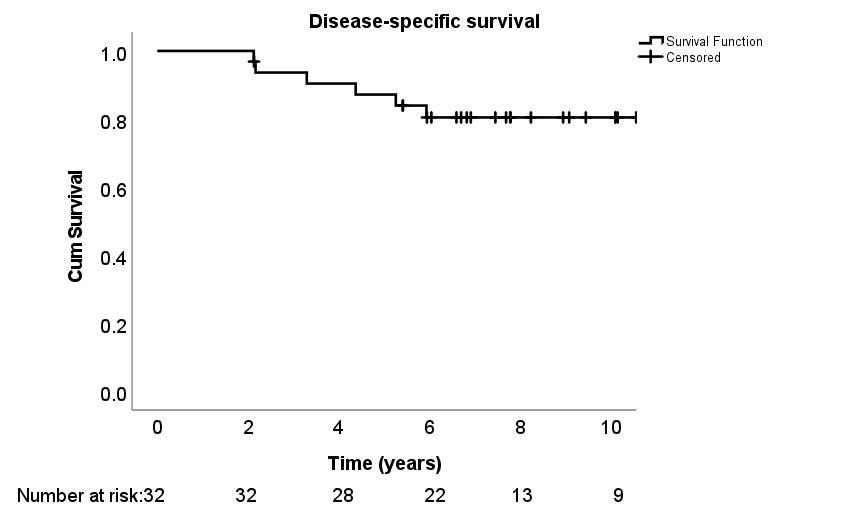

Supplement: Supplementary file 4 — Supplementary file4 (JPG 23 kb) [file 10434_2023_13312_MOESM4_ESM.jpg]
