# Supplementary material for: Patterns of Metastatic Recurrence of Genetically Confirmed Myxoid Liposarcoma
Source: Ann Surg Oncol. 2023 Mar 12;30(7):4489–97. doi: 10.1245/s10434-023-13312-x (PMC10250512; doi:10.1245/s10434-023-13312-x)
Supplement: Supplementary file 5 — Supplementary file5 (DOCX 11 kb) [file 10434_2023_13312_MOESM5_ESM.docx]

**ELECTRONIC SUPPLEMENTARY MATERIAL 1** PRISMA flow diagram of the systematic review for locations of myoxid liposarcoma metastases. Chart based on [25]. *PRISMA* Preferred Reporting Items for Systematic Reviews and Meta-Analyses

**ELECTRONIC SUPPLEMENTARY MATERIAL 2** Local disease progression-free survival of patients with myxoid liposarcoma in the current series

**ELECTRONIC SUPPLEMENTARY** **3**. Disease-free survival of patients with myxoid liposarcoma in the current series

**ELECTRONIC SUPPLEMENTARY 4** Disease-specific survival of patients with myxoid liposarcoma in the current series
